# Double burden of malnutrition and its socio-demographic determinants among children and adolescents in Malaysia: National Health And Morbidity Survey 2019

**DOI:** 10.1186/s41043-024-00583-7

**Published:** 2024-06-24

**Authors:** Wai Kent Lai, Lalitha Palaniveloo, Syafinaz Mohd Sallehuddin, Shubash Shander Ganapathy

**Affiliations:** 1 Institute for Public Health, National Institutes of Health, Ministry of Health, Shah Alam, Malaysia; 2 Raub Health District Office, Pahang State Health Deparment, Ministry of Health, Raub, Malaysia

**Keywords:** Double burden, Malnutrition, Children, Adolescent, Anthropometric assessment, Socio-demographic

## Abstract

**Introduction:**

Malaysia faces the threat of a double burden of malnutrition where undernutrition and overweight (including obesity) coexist in the same population. This study aimed to determine the anthropometric assessment among children and adolescents aged 5 to 17 years and its association with socio-demographic factors.

**Methods:**

Data were extracted from the National Health and Morbidity Survey conducted in 2019. This cross-sectional survey applied a two-stage stratified sampling design. Socio-demographic characteristics were obtained. Weight and height were measured, age- and sex-specific standard scores for height and BMI were calculated to establish individual’s anthropometric assessment. Having either stunting or thinness was considered undernutrition, while being overweight (including obesity) was considered overnutrition. If someone had undernutrition and/or overnutrition, they were classified as having malnutrition. The prevalence was determined using complex sampling analysis, while the association was assessed through logistic regression. The analysis included a total of 3,185 respondents.

**Results:**

The prevalence of stunting, thinness, overweight and obesity among the respondents aged 5 to 17 years was 12.7%, 10.0%, 15.0% and 14.8%, respectively. The overall prevalence of malnutrition was 48.3%. Respondents residing in rural had 1.35 times more likelihood of experiencing undernutrition [AOR = 1.35, 95% CI (1.04, 1.77)] compared to their urban counterparts. Boys exhibited a greater likelihood of being overweight and obese than girls [AOR = 1.40, 95% CI (1.13, 1.73)]. Respondents aged 10 to 14 years were 1.37 times more likely to be overnutrition than those aged 5 to 9 years old [AOR = 1.37, 95% CI (1.09, 1.73)].

**Conclusion:**

There is growing evidence of the increasing prevalence of coexistence of undernutrition along with overweight and obesity among children and adolescents in Malaysia. Moving forward, greater initiatives and efforts are required to formulate strategies for planning and implementing programs and policies to expedite progress in improving nutrition.

## Introduction

The analysis of the dynamics of undernutrition and obesity indicates that the substantial double burden of malnutrition (DBM) is propelled by the rapid rise in overweight and obesity prevalence occurring in low- and middle-income countries (LMICs), which are simultaneously undergoing a slower reduction in the prevalence of undernutrition [1]. The prevalence of DBM among adolescents is widespread in LMICs, especially at the population level [2]. A pooled analysis of 2,181 population-based studies revealed significant variability in the height and body mass index (BMI) trajectories of school-aged children and adolescents over age and time across various countries and territories [3]. The report highlighted changes in late-adolescence BMI in Malaysia from 1985 to 2019, indicating a noteworthy rise of over 3 kg/m^2^ in both genders. Notably, Malaysian boys exhibited unfavourable changes such as insufficient height gain, excessive weight gain or a combination of both, in comparison to their counterparts in other countries. At the same time, report from UNICEF indicated that Malaysia is confronted with the risk of experiencing the threat of a DBM [4].

Historically, malnutrition has been studied and addressed in separate categories, with one focus on chronic or acute undernutrition, energy inadequacy and micronutrient deficiencies, while the other concentrates on overweight, obesity and dietary excess [5]. The World Health Organization (WHO) has characterized the DBM as the simultaneous occurrence of undernutrition along with overweight and obesity, or diet-related non-communicable diseases in individuals, households and populations, and spanning various life stages [6]. Nutrition is one of the most critical factors affecting the onset and progression of pubertal development [7]. Nutritional status during childhood has a significant effect on pubertal development. Some of the critical indicators of the nutritional status of a given population are based on anthropometric data such as BMI for adults, while weight-for-age z-scores (WAZ), length or height-for-age z-scores (LAZ or HAZ), weight-for-height z-scores (WHZ), and BMI-for-age z-scores (BAZ) for children and adolescents.

Child malnutrition is a worldwide challenge with profound implications for survival rates, the incidence of acute and chronic illnesses, healthy development, and the economic productivity of both individuals and communities [8]. Malnutrition is a disorder affecting multiple systems. In severe cases, it can compromise immunity, prolong wound healing, and elevate morbidity and mortality rates during medical procedures. Furthermore, malnutrition worsens the outcome of the illnesses, for instance, respiratory muscle weakness may impede a child’s ability to be weaned from mechanical ventilation. In addition, persistent and severe malnutrition can lead to a permanent delay in intellectual development [9]. Therefore, recognizing this double burden should reveal opportunities to address both issues simultaneously as they harm health throughout the course of life.

The impact of undernutrition experienced in the past 4 to 5 decades will continue to influence our health for many future years [1]. For instance, stunting experienced in previous decades will have substantial effects decades later, leading to increased visceral fat and greater risks to major non-communicable diseases [5]. Child stunting has various immediate and prolonged effects, including increased rates of morbidity and mortality, poor child development and learning capacity, increased susceptibility to infections, reduced fat oxidation, diminished energy expenditure, insulin resistance, decreased working capacity and adverse maternal reproductive outcomes in adulthood [10]. On the other hand, overweight and obesity during childhood frequently endure into adulthood, and elevating the likelihood of encountering non-communicable diseases at a younger age [11].

Nutritional assessment and management in children over the age of 5 are comparatively less emphasized and addressed than in infants and children under 5 years old. Further attention and efforts are needed for school-age children and adolescents as they are also susceptible to the impact of malnutrition. Considerable research and routine surveillance are required to investigate the extent to which the double burden of malnutrition exists in this particular age group. This effort could potentially revising important policy decisions. Therefore, this study aimed to determine the magnitude of the double burden of malnutrition and to examine its socio-demographic associations among children aged 5 to 17 years using nationally representative samples in Malaysia. The results from this study were also compared with the findings on the anthropometric assessment of the respective age groups in the NHMS conducted in 2015.

## Methods

### Sampling design and sample size

In Malaysia, the National Health and Morbidity Survey (NHMS) is conducted to obtain community-based information on the distribution of health problems and the health needs of the people. This information is essential for the Ministry of Health to examine its priorities and programs, plan for future budget allocation, and assess the effectiveness of the current strategies. The NHMS, which was conducted in 2019, focused on the status of non-communicable diseases in Malaysia, and anthropometric assessment was part of the survey.

This study utilized secondary data from the National Health and Morbidity Survey (NHMS), conducted by the Institute for Public Health, Ministry of Health Malaysia, in 2019. This cross-sectional survey aimed to determine the prevalence of non-communicable diseases, healthcare demand and health literacy in Malaysia [12]. This survey employed two-stage stratified random sampling to ensure the national representativeness of the sample. The general population was stratified by 13 states and three federal territories, followed by urban and rural strata within the states and federal territories. In the initial stage, Enumeration Blocks (EB) were systematically sampled within each stratum, followed by the random selection of 12 living quarters (LQ) within each chosen EB. A total of 475 EBs were selected, encompassing approximately 5,676 LQs in the survey. All individuals residing in the selected LQs for a minimum of two weeks before data collection were eligible to participate in this survey. A detailed methodology and sampling design of the study is available in the NHMS 2019 technical report [12].

The sample size was calculated by comparing two proportions, where the prevalence of obesity among children aged below 18 years old, between boys (13.6%) and girls (10.0%) in NHMS 2015 [13], at a significance level (α) of 0.05 and 80% study power. The required sample size was 2,520. It was adjusted to 3,877 after accounting for a 35% non-response rate.

In this study, individuals below the age of 5 and those above 17 were not considered (*n* = 11,383), since the study specifically targeted the population of children aged 5 to 17 years. Respondents with incomplete socio-demographic and nutritional status data were excluded (*n* = 397). Following these exclusions, a total of 3,185 respondents were included in the current analyses. The estimated population for children aged 5 to 17 years obtained in this study closely resembles the actual population structure of Malaysia for 2019, as projected by the Department of Statistics Malaysia [14], suggesting the sample size is representative.

### Data collection

A training workshop for data collectors, nurses, and field supervisors was conducted before data collection in July 2019. The main goals of the training were to acquaint the data collection teams with the questionnaires, to develop and improve interpersonal skills, and to recognize the value of effective teamwork. Data collection started in July 2019 and continued through October 2019, covering all states and federal territories in Malaysia. The data were transmitted to the Institute for Public Health for quality control and dataset management.

### Ethical considerations

**Ethical approval** was granted by the Medical Research and Ethics Committee, Ministry of Health Malaysia, under the reference number NMRR-18-3085-44207. Before data collection, written informed consents were obtained from the participants or guardians, with an additional assent form signed by participants between seven and 18 years of age.

### Socio-demographic characteristics

A structured questionnaire was used to collect socio-demographic characteristics such as sex, age, ethnicity, residing location and household income. Face-to-face interviews were conducted for children aged 13 years and above, while for children below 13 years, the parent or guardian responded to the interview on their behalf. Age was recorded as a continuous variable and then transformed into three categories, namely 5–9 years, 10–14 years, and 15–17 years. Residing location was either urban or rural and ethnicity was categorized into Malay, Chinese, Indian and others. Information on household income was cross-referenced with parental interviews and categorized into three groups: the bottom 40% (B40) representing low income, the middle 40% (M40) representing middle income, and the top 20% (T20) representing high income. These categories were determined based on the cut-off values from the Department of Statistics Malaysia.

### Anthropometric measurements

Body weight and height were measured by trained personnel using Tanita Personal Scale HD 319 to the nearest 0.1 kg and SECA Stadiometer 213 to the nearest 0.1 cm, respectively. Both tools had been validated and calibrated before the survey. Each participant underwent two measurements to obtain an average value. The anthropometric assessment was determined based on the WHO Growth Reference for 5–19 years [15]. Height-for-age z-scores (HAZ) and BMI-for-age z-scores (BAZ) were computed through WHO AnthroPlus software [16]. A child was defined as stunted (short stature for age) and thin if their height-for-age and BMI-for-age indices were below two standard deviations (SD) of the WHO reference population median [15]. On the other hand, a child was considered overweight and obese if their BMI-for-age indices were more than one SD and more than two SD, respectively.

Any one or more of the two conditions, stunting, or thinness, was defined as undernutrition; overweight, or obesity, was defined as overnutrition. Malnutrition is defined as the coexistence of both undernutrition and overnutrition in the same population across the life course [6]. Therefore, in this study, if someone had undernutrition and/or overnutrition, they were classified as having malnutrition.

### Statistical analysis

Data were entered into and analyzed with the IBM SPSS Statistics 26. Complex sample analysis procedures were used in the analysis. Each respondent was assigned a weighting factor to account for non-response and the diverse probabilities of selection. Weighted descriptive statistics from the survey were calculated to furnish estimates that are representative at the national level. An analysis of socio-demographic factors associated with nutritional status was conducted. Counts and percentages were utilized to depict categorical variables.

Each prevalence of malnutrition, i.e., stunting, thinness, wasting, overweight, obese and the total prevalence of undernutrition, overnutrition and malnutrition were determined. A chi-square test was performed to determine bivariate associations between socio-demographic factors and anthropometric assessment of children and adolescents. Row percentage was reported for the proportion of respondents from each socio-demographic characteristics in the respective anthropometric assessment, while column percentage was shown for the total proportion of respondents in each socio-demographic characteristic. Furthermore, binomial logistic regression was employed to determine the relationship between socio-demographic factors and the anthropometric assessment of children and adolescents. The results of logistics regression models were presented as odds ratio (OR). The confidence level was established at 95%, and the significance level was determined with a p-value of less than 0.05. Simple logistic regression was applied to calculate the crude OR, and variables exhibiting a p-value less than 0.25 were included in the multivariable logistic regression model. Multicollinearity and interaction terms were examined. The classification table and Nagelkerke R^2^ for each model were reported. The conclusive model was presented with adjusted odds ratio (AOR).

## Results

### Descriptive characteristics of participants

A total of 3,185 children and adolescents were investigated. As shown in Table 1 and 51.0% were boys and 49.0% were girls. More than half of them were Malays (61.2%), followed by 15.1% of Chinese and 13.5% of Bumiputera Sabah and Sarawak. Over half of the respondents were from lower-income groups (67.2%). About one-quarter of the respondents lived in rural areas.

### Double burden of undernutrition and overnutrition

Among the studied population, the prevalence of stunting and thinness were 12.7% and 10.0%, respectively (Table 1); overall, the prevalence of undernutrition was 21.0% (Table 2). In addition, the prevalence of concurrent stunting and thinness was 1.6% (estimated population = 103,781; 95% CI: 1.08, 2.40). On the other hand, the prevalence of overweight and obesity was 15.0% and 14.8%, respectively, which made up 29.8% of the overall prevalence of overnutrition. Moreover, the prevalence of concurrent stunting and overweight (including obesity) was 2.3% (estimated population = 148,534; 95% CI: 1.69, 3.13). Thus, the overall prevalence of malnutrition was 48.3% (Table 2).

### Factors associated with stunting among children and adolescents

The stunting rate differed statistically among age groups and residing areas (Table 1). In simple logistic analysis as shown in Table 3, Chinese seems less likely to be stunted than Malays [COR = 0.47, 95% CI (0.23, 0.98), *p* = 0.040]. Participants living in rural areas were 1.62 times more likely to be stunted than urban participants [COR = 1.62, 95% CI (1.14, 2.31), *p* = 0.007]. However, neither variable was significant in multiple logistic regression. The results also revealed that the percentage of participants reporting stunted growth showed no significant difference across gender, age group and household income.

### Factors associated with thinness among children and adolescents

The rate of thinness was not statistically different among all socio-demographic characteristics (Table 1). Table 3 shows that Indians were 2.60 times more likely to be thin as compared to Malays [COR = 2.60, 95% CI (1.14, 2.31), *p* = 0.007]. The findings also revealed that the percentage of participants reporting thinness did not vary based on gender, age group, residing area and household income. Multiple logistic regression was not performed as only one factor was significant and the p-values of other factors were more than 0.25 in simple logistic regression.

### Factors associated with obesity among children and adolescents

The prevalence of obesity was statistically different among boys and girls (Table 1). In the simple logistic regression shown in Table 3, boys were 1.55 times more likely to be obese as compared to females [COR = 1.55, 95% CI (1.21, 1.99), *p* < 0.001]. Participants aged 10 to 14 years were 1.37 times more likely to be obese compared to those aged 5 to 9 [COR = 1.37, 95% CI (1.03, 1.83), *p* = 0.028]. Both variables remained significant in multiple logistic regression, where boys were more likely to be obese as compared to girls [AOR = 1.52, 95% CI (1.18, 1.96), *p* = 0.001] and adolescents aged 10 to 14 years were more like to be obese as compared to children aged 5 to 9 [AOR = 1.37, 95% CI (1.02, 1.85), *p* = 0.038]. The results also indicated that the proportion of children and adolescents reporting obesity showed no significant difference across ethnicity groups, residing area and household income (Table 4).

### Factors associated with undernutrition among children and adolescents

The rate of undernutrition was statistically different among different age groups, ethnicities and residing areas (Table 2). In simple logistic analysis as shown in Table 3, Chinese seem less likely to be undernourished than Malays [COR = 0.49, 95% CI (0.28, 0.86), *p* = 0.013]. Participants living in rural areas were 1.45 times more likely to be stunted than those living in urban areas [COR = 1.45, 95% CI (1.06, 1.97), *p* = 0.019]. Both variables remained significant in multiple logistic regression, where Chinese were less likely to be undernutrition as compared to Malays [AOR = 0.53, 95% CI (0.29, 0.94), *p* = 0.029], and participants living in rural areas were more likely to be undernutrition as compared to those living in urban areas [AOR = 1.35, 95% CI (1.04, 1.77), *p* = 0.027]. The findings also revealed that the percentage of participants reporting undernutrition did not vary by gender, age group and household income.

### Factors associated with overnutrition among children and adolescents

The prevalence of overnutrition was statistically different among different genders (Table 2). In simple logistic analysis as shown in Table 3, boys were 1.39 times more likely to be overnutrition than females [COR = 1.39, 95% CI (1.13, 1.72), *p* = 0.002]. Participants aged 10 to 14 years were 1.37 times more likely to be overnutrition compared to those aged 5 to 9 years [COR = 1.37, 95% CI (1.09, 1.72), *p* = 0.008]. Both variables remained significant in multiple logistic regression, where boys were more likely to be obese as compared to girls [AOR = 1.40, 95% CI (1.13, 1.73), *p* = 0.002] and adolescents aged 10 to 14 years were more likely to be obese as compared to children aged 5 to 9 [AOR = 1.37, 95% CI (1.09, 1.73), *p* = 0.007]. The results also revealed that the percentage of children and adolescents reporting overnutrition showed no significant difference based on ethnicity group, residing area and household income.

**Table 1:** Socio-demographic characteristics among children aged 5 to 17 years by anthropometric assessment in Malaysia, 2019

Variables	Stunting (HAZ < -2SD)				Thinness (BAZ < -2SD)				Overweight (1SD < BAZ ≤ 2SD)				Obese (BAZ > 2SD)			
	*n*	Est. pop.	(%)	*p*	*n*	Est. pop.	(%)	*p*	*n*	Est. pop.	(%)	*p*	*n*	Est. pop.	(%)	*p*
**Gender**				0.787				0.901				0.400				**< 0.001**
Male	195	410,784	(12.5)		153	329,327	(10.1)		229	511,438	(15.7)		270	569,129	(17.5)	
Female	201	407,644	(12.9)		129	311,037	(9.9)		227	447,866	(14.2)		194	378,056	(12.0)	
**Age group**				**0.033**				0.912				0.331				0.096
5–9	166	299,588	(12.7)		122	230,079	(9.9)		158	306,720	(13.2)		176	308,181	(13.3)	
10–14	131	239,464	(10.2)		110	226,066	(9.6)		198	366,861	(15.6)		207	407,915	(17.4)	
15–17	99	279,376	(16.0)		50	184,219	(10.6)		100	285,722	(16.4)		81	231,089	(13.3)	
**Ethnicity**				0.092				0.070				0.383				0.670
Malay	286	526,001	(13.4)		210	395,280	(10.1)		316	577,447	(14.8)		308	593,989	(15.2)	
Chinese	18	65,380	(6.7)		14	57,614	(5.9)		44	181,985	(18.8)		38	112,269	(11.6)	
Indian	15	27,651	(8.1)		27	76,989	(22.7)		25	55,237	(16.3)		28	56,926	(16.8)	
Other *Bumiputera*	56	129,567	(14.9)		20	54,147	(6.3)		60	113,312	(13.1)		74	139,054	(16.1)	
Others	21	69,829	(21.7)		11	56,334	(17.5)		11	31,322	(9.7)		16	44,947	(14.0)	
**Residing area**				**0.007**				0.536				0.329				0.196
Urban	218	542,421	(11.3)		174	459,297	(9.6)		283	737,707	(15.4)		296	734,149	(15.3)	
Rural	178	276,006	(17.1)		108	181,067	(11.2)		173	221,597	(13.7)		168	213,037	(13.2)	
**Household income**				0.853				0.900				0.455				0.308
Bottom 40%	268	514,363	(12.8)		179	389,704	(9.8)		296	612,480	(15.4)		299	621,786	(15.6)	
Middle 40%	74	167,553	(11.6)		59	148,651	(10.3)		106	220,596	(15.3)		96	186,324	(12.9)	
Top 20%	29	66,144	(13.1)		29	59,159	(11.8)		30	56,677	(11.3)		38	89,491	(17.8)	
**Total**	396	818,428	(12.7)		282	640,364	(10.0)		456	959,304	(15.0)		464	947,185	(14.8)	

n = unweighted count; Est. pop. = estimated population

**Table 2:** Socio-demographic characteristics among children aged 5 to 17 years by malnutrition groups in Malaysia, 2019

Variables	Undernutrition (HAZ < -2SD or BAZ < -2SD)				Overnutrition (BAZ > 1SD)				Malnutrition		
	*n*	Est. pop.	(%)	*p*	*n*	Est. pop.	(%)	*p*	*n*	Est. pop.	(%)
**Gender**				0.855				**0.002**			
Male	324	697,509	(21.2)		499	1,080,566	(33.2)		780	1,700,978	(51.7)
Female	301	657,502	(20.8)		421	825,923	(26.2)		684	1,411,988	(44.7)
**Age group**				**0.022**				0.051			
5–9	268	493,960	(21.0)		334	614,902	(26.5)		575	1,064,281	(45.1)
10–14	214	413,343	(17.6)		405	774,777	(33.0)		585	1,143,676	(48.8)
15–17	143	447,708	(25.7)		181	516,811	(29.7)		304	905,010	(52.0)
**Ethnicity**				**0.012**				0.830			
Malay	456	849,769	(21.5)		624	1,171,435	(30.0)		1017	1,918,028	(48.6)
Chinese	30	114,094	(11.8)		82	294,254	(30.3)		106	397,605	(41.0)
Indian	39	99,014	(29.1)		53	112,163	(33.0)		90	208,594	(61.4)
Other Bumiputera	70	169,771	(19.5)		134	252,366	(29.2)		197	402,216	(46.3)
Others	30	122,363	(38.1)		27	76,270	(23.7)		54	186,524	(58.1)
**Residing area**				**0.018**				0.082			
Urban	363	935,437	(19.4)		579	1,471,856	(30.7)		896	2,303,360	(47.8)
Rural	262	419,574	(25.9)		341	434,633	(26.9)		568	809,606	(49.9)
**Household income**				0.999				0.596			
Bottom 40%	417	839,045	(20.9)		595	1,234,266	(31.0)		956	1,985,123	(49.5)
Middle 40%	123	301,702	(20.8)		202	406,920	(28.1)		306	666,680	(45.9)
Top 20%	47	104,847	(20.7)		68	146,168	(29.1)		111	240,161	(47.4)
**Total**	625	1,355,011	(21.0)		920	1,906,489	(29.8)		1464	3,112,967	(48.3)

n = unweighted count; Est. pop. = estimated population

**Table 3:** Logistic regression analysis of socio-demographic factors associated with stunting, thinness and obesity

Variables	Stunting				Thinness		Obesity			
	Crude OR (95% CI)	*p*	Adjusted OR (95% CI)^a^	*p*	Crude OR (95% CI)^b^	*p*	Crude OR (95% CI)	*p*	Adjusted OR (95% CI)^c^	*p*
**Gender**										
Male	0.96 (0.73,1.28)	0.787			1.03 (0.69,1.52)	0.901	1.55 (1.21,1.99)	**< 0.001**	1.52 (1.18,1.96)	**0.001**
Female	Reference				Reference		Reference		Reference	
**Age group**										
5–9	Reference		Reference		Reference		Reference		Reference	
10–14	0.78 (0.56,1.08)	0.139	0.80 (0.58,1.10)	0.171	0.97 (0.64,1.46)	0.879	1.37 (1.03,1.83)	**0.028**	1.37 (1.02,1.85)	**0.038**
15–17	1.31 (0.89,1.92)	0.167	1.27 (0.90,1.78)	0.175	1.07 (0.58,2.00)	0.823	1.00 (0.66,1.50)	0.992	1.06 (0.70,1.62)	0.779
**Ethnicity**										
Malay	Reference		Reference		Reference		Reference			
Chinese	0.47 (0.23,0.98)	**0.040**	0.51 (0.24,1.07)	0.075	0.56 (0.28,1.13)	0.104	0.73 (0.42,1.28)	0.269		
Indian	0.58 (0.26,1.30)	0.182	0.60 (0.26,1.39)	0.234	2.60 (1.20,5.66)	**0.016**	1.12 (0.65,1.94)	0.677		
Other Bumiputera	1.14 (0.76,1.71)	0.528	1.07 (0.72,1.58)	0.751	0.59 (0.30,1.17)	0.131	1.07 (0.75,1.53)	0.712		
Others	1.80 (0.61,5.32)	0.285	1.56 (0.56,4.31)	0.393	1.89 (0.41,8.69)	0.413	0.91 (0.41,2.00)	0.810		
**Residing area**										
Urban	Reference		Reference		Reference		1.19 (0.91,1.55)	0.197	1.19 (0.90,1.57)	0.228
Rural	1.62 (1.14,2.31)	**0.007**	1.39 (0.99,1.93)	0.055	1.19 (0.68,2.08)	0.536	Reference		Reference	
**Household income**										
Bottom 40%	Reference				Reference		Reference		Reference	
Middle 40%	0.89 (0.58,1.36)	0.584			1.06 (0.54,2.07)	0.871	0.80 (0.56,1.15)	0.233	0.82 (0.57,1.18)	0.274
Top 20%	1.02 (0.53,1.95)	0.948			1.23 (0.52,2.94)	0.636	1.17 (0.74,1.87)	0.499	1.16 (0.73,1.85)	0.533

^a^ Classification table (overall correctly classified percentage) = 87.3%; Nagelkerke R^2^ = 3.1%. ^b^ Multivariable logistic regression was not performed as only one variable having p-value less than 0.25 from the univariate analysis. ^c^ Classification table (overall correctly classified percentage) = 84.9%; Nagelkerke R^2^ = 1.8%

**Table 4:** Logistic regression analysis of socio-demographic factors associated with undernutrition and overnutrition

Variables	Undernutrition				Overnutrition				
	Crude OR (95% CI)	*p*	Adjusted OR (95% CI)^a^	*p*		Crude OR (95% CI)	*p*	Adjusted OR (95% CI)^b^	*p*
Gender									
**Male**	1.02 (0.79,1.32)	0.855				1.39 (1.13,1.72)	**0.002**	1.40 (1.13,1.73)	**0.002**
Female	Reference					Reference		Reference	
**Age group**									
5–9	Reference		Reference			Reference		Reference	
10–14	0.81 (0.62,1.06)	0.121	0.83 (0.64,1.09)	0.174		1.37 (1.09,1.72)	**0.008**	1.37 (1.09,1.73)	**0.007**
15–17	1.31 (0.92,1.86)	0.141	1.23 (0.91,1.66)	0.188		1.17 (0.89,1.54)	0.270	1.19 (0.90,1.57)	0.228
**Ethnicity**									
Malay	Reference		Reference			Reference			
Chinese	0.49 (0.28,0.86)	**0.013**	0.53 (0.29,0.94)	**0.029**		1.02 (0.68,1.52)	0.935		
Indian	1.50 (0.78,2.89)	0.228	1.57 (0.80,3.07)	0.186		1.15 (0.66,2.00)	0.616		
Other Bumiputera	0.88 (0.61,1.29)	0.523	0.83 (0.57,1.21)	0.332		0.96 (0.74,1.26)	0.782		
Others	2.24 (0.90,5.60)	0.084	1.98 (0.89,4.43)	0.096		0.73 (0.37,1.45)	0.366		
**Residing area**									
Urban	Reference		Reference			1.20 (0.98,1.48)	0.082	1.21 (0.98,1.49)	0.079
Rural	1.45 (1.06,1.97)	**0.019**	1.35 (1.04,1.77)	**0.027**		Reference		Reference	
**Household income**									
Bottom 40%	Reference					Reference			
Middle 40%	0.99 (0.65,1.51)	0.971				0.87 (0.65,1.18)	0.379		
Top 20%	0.99 (0.53,1.85)	0.970				0.92 (0.63,1.37)	0.646		

^a^ Classification table (overall correctly classified percentage) = 79.0%; Nagelkerke R^2^ = 3.9%. ^b^ Classification table (overall correctly classified percentage) = 70.2%; Nagelkerke R^2^ = 1.5%

## Discussion

The findings of the study are consistent with the double burden of malnutrition in other Malaysian population studies [17–25]. From this study, overweight and obesity as a form of malnutrition are more prevalent than thinness and stunting among children and adolescents aged 5 to 17 years in Malaysia. Another set of secondary data from the NHMS conducted in 2015 was computed to describe the prevalence of the anthropometric assessments for the same age group. The analysis revealed an upward trend in all forms of malnutrition between 2015 and 2019. Specifically, there were notable rises in the rates of stunting, thinness, overweight, and obesity. This led to an increase in both undernutrition and overnutrition, resulting in a higher overall malnutrition rate. Given the high overweight and obesity prevalence observed among children and adolescents in the study population, the results provide further support for the need for continued research and programmatic focus on the double burden of malnutrition throughout childhood in Malaysia.

The prevalence of overweight and obesity in Malaysia was higher than the regional prevalence in East Asia and Pacific, which was 23% [4]. This prevalence was also higher than in neighboring countries within the WHO South-East Asia Region, where the prevalence of overweight and obesity in the region was 12.1% and 6.1%, respectively [26]. The global prevalence of obesity among children and adolescents in 2016 was 5.6% in girls and 7.8% in boys [27]. The study revealed that the prevalence of obesity in the country was double the global prevalence for both genders. Analyses of worldwide data regarding overweight and obesity prevalence determined that the increase in excess weight among children and adolescents has stabilized in high-income countries but persists in LMICs [27, 28].

The DBM is often associated with nutrition transition in the country [29]. The surge in overweight is primarily attributed to very rapid transformations in the food system, particularly the widespread availability of affordable ultra-processed food and beverages in LMICs, along with substantial declines in physical activity across various domains, including work, transportation, home, and leisure, owing to the introductions of activity-saving technologies [6]. Furthermore, the nutrition transition in Malaysia was attributed by three primary domains, namely the food environment (including shifts in the types of foods consumed), changes in lifestyle and behavior (including sedentary urban living, increased reliance on eating out and the influence of trendy food culture) and government policies (involving nutrition action plans, subsidies and taxes) [30].

On the other hand, the global prevalence of thinness among children and adolescents aged 5 to 19 years was 8.4% for girls and 12.4% for boys in 2016 [27]. Findings from this study showed that the overall prevalence of thinness in Malaysia (10.0%) was lower than the global prevalence. However, it was higher than the East Asia and Pacific region (6%) in UNICEF reports [4]. Compared to other neighboring countries in the WHO South-East Asia Region, the prevalence of thinness in the region (10.8%) was comparable to that in Malaysia [26]. In terms of stunting, the finding from the current study was lower than the prevalence in a local research, which was 16.5% [21]. The study used slightly different age groups (six to 19 years) and was limited to only one district which was not generalizable to the national prevalence. The prevalence of stunting in the current study was much lower than in the WHO South-East Asia Region, which was 30.1% [26].

The associations between anthropometric assessment and socio-demographic variables varied. The present study demonstrated an association between boys and higher odds of overnutrition (inclusive of overweight and obesity). This could be due to the sex differences in overweight or obesity on body weight perceptions and body image. This finding was consistent with previous works [20, 31]. However, it was in contrast with studies demonstrating females were more likely to be overweight and obese compared to males [32, 33]. In this study, participants aged 10 to 14 years were more likely to be overweight and obese as compared to those aged 5 to 9 years. This could be due to increased access to food at an older age. No evidence of associations between ethnicity, residing area, household income and child overweight (including obesity) were found in this study. The present study reported that the prevalence of overweight and obesity was higher in urban area compared to rural area. However, the difference was not significant. Other studies reported that children and adolescents living in urban areas were more likely to be overweight or obese compared to those living in rural areas [31, 32].

On the other hand, the present study reported no gender and age group differences in the odds of being stunted, thin and undernutrition. In another study, females were observed to be more stunted but less thinner than males in most South East Asian countries across time [26]. Some studies demonstrated a higher prevalence of male stunting [34, 35]. A local study reported that stunting risk increased by 8% for every one-year increase in age [20]. In addition, boys were more likely to be thin compared to girls [20, 33, 35]. Thinness prevalence was also slightly lower among children aged 10 to 14 and 15 to 19 years as compared to those aged six to nine years [20], while another study showed that early adolescence (10 to 13 years old) was significantly associated with thinness [35].

Findings from this study unveiled the urban-rural differences in undernutrition but did not show such disparities in overnutrition. Participants residing in rural areas exhibited a higher likelihood of experiencing undernutrition in this study. This could possibly be attributable to the superior socioeconomic development in urban areas compared to rural areas. The associations between residing location and undernutrition observed in the study are consistent with previous research. For instance, living in rural areas was significantly associated with stunting [31, 35].

In this study, the findings indicated that the proportion of any nutritional status did not show a significantly difference based on household income. In comparison with other studies, children and adolescents from low household income were more likely to be stunted and underweight [26, 32, 34, 35]. In contrast, middle and high household income were significantly associated with overweight and obesity [26, 32].

Factors contributing to undernutrition and overnutrition are complex and multifaceted, posing challenges in understanding the conditions and developing effective interventions and policies. Distinct communities, policies, programs, governance structures, and funding channels usually manage actions to address various types of malnutrition. Some evidence suggests that initiatives targeting undernutrition have unintentionally increased the risks of obesity and diet-related non-communicable diseases in LMICs undergoing rapid changes in food environments [36].

Several key implications emerge from this study. Since 2015, Malaysia has seen a rise in the prevalence of stunting, thinness, overweight and obesity, led to an increase in the combined burden of these conditions. Transition to overnutrition dominance was also getting apparent in children and adolescents. Considering that the shift from underweight to obesity can happen quickly and that both conditions share common drivers like early life nutrition, diet quality, food environments, and socioeconomic factors [37], approach to double-duty actions by simultaneously addressing both undernutrition and issues related to overweight, obesity, and diet-related non-communicable diseases should be initiated [36]. Accordingly, addressing the double burden of malnutrition requires coordinated efforts across multiple sectors, including education, healthcare, and community engagement. For instance, education initiatives can increase knowledge and awareness about healthy eating habits and the importance of balanced nutrition. Healthcare providers play a crucial role in early detection and treatment of both undernutrition and overnutrition. Community engagement can enhance access to fresh, nutritious foods through local food systems such as community gardens, farmers’ markets and local food cooperatives. This collective effort fosters a supportive environment, making it easier for individuals to maintain healthy lifestyles.

Malaysia has implemented various multi-sectoral nutrition programs to tackle malnutrition, as indicated in the National Plan of Action for Nutrition of Malaysia III [38]. Key initiatives include educational programs, school-based interventions, and government guidelines. For instance, monitoring of standard menus in childcare centres, monitoring of foods sold in school canteens in consistence with the School Canteen Management Guidelines [39], and monitoring the compliance of standard menus in boarding schools are parts of the promoting and advocating healthy eating activities. Continuous efforts are needed to enhance adherence to these guidelines, aiming to foster healthy eating habits among school-going children. Furthermore, Nutritious School Meal Programme was launched and aimed at providing healthy meals to primary school children [40]. This program focuses on offering balanced meals that meet the caloric and nutritional needs of students, including food, beverages, and fruits provided during school breaks. However, this program are only available in selected schools and not expanded to secondary schools, yet their effectiveness has not been evaluated. In addition, an updated version of Malaysian Dietary Guideline for Children and Adolescents was developed to provide specific recommendations on knowledge, attitude and practices related to food and nutrition, focusing on children and adolescents in Malaysia [41].

One of the limitations of this study was the lack of clinical diagnosis of malnutrition. It should consider other relevant factors additional to anthropometry, such as body composition, other signs of clinical undernutrition, or potential genetic syndromes. Besides, malnutrition in terms of micronutrients was not included in the present study. Micronutrient malnutrition is recognized as a component of undernutrition. However, the present study was unable to include this form of undernutrition in the DBM estimates. In addition, this study employed a cross-sectional design, which was unable to determine the causal inference relationship. Other potential correlates, such as parental education level, parental nutritional status, breastfeeding status of the child, family size, food security and dietary diversity were not included in this study.

## Conclusion

This survey suggests that undernutrition is predominantly in rural areas compared to urban areas. The prevalence of overnutrition was significantly higher among males and those aged 10 to 14 years than their counterparts. There is growing evidence of the increasing prevalence of coexistence of undernutrition along with overweight and obesity among children and adolescents in Malaysia. Therefore, there is a crucial need to address the growing double burden of malnutrition (undernutrition, overweight and obesity) in Malaysia. Multisectoral approaches involving education, healthcare, and community engagement are essential to tackle the double burden of malnutrition effectively. Looking forward, greater initiatives and efforts are required to develop strategies for planning and implementing programs and policies to accelerate progress in improving nutrition. National nutrition and health programs focusing on malnutrition should be extended to children and adolescents in school in order to consolidate healthy growth through the entire developmental period.

## Data Availability

The data that support the findings of this study are available from Ministry of Health Malaysia but restrictions apply to the availability of these data, which were used under license for the current study, and so are not publicly available. Data are, however, available from the authors upon reasonable request and with permission of Ministry of Health Malaysia.
